# Impact of COVID-19 on Antenatal Care Utilization Among Pregnant Women in Qassim, Saudi Arabia

**DOI:** 10.7759/cureus.19554

**Published:** 2021-11-14

**Authors:** Unaib Rabbani, Abdullah A Saigul, Amel Sulaiman, Tayseer H Ibrahim

**Affiliations:** 1 Family Medicine Academy, Qassim Health Cluster, Buraidah, SAU; 2 Academic Affairs and Training Center, Maternity and Children Hospital, Buraidah, SAU

**Keywords:** women health, utilization, saudi arabia, pregnancy, covid-19, antenatal care

## Abstract

Background and objectives

Coronavirus disease 2019 (COVID-19) pandemic has affected routine service delivery which might affect antenatal care (ANC) utilization among pregnant women. This study aimed to assess the proportion of missed appointments among pregnant women in the Qassim region of Saudi Arabia during the COVID-19 pandemic.

Methods

A facility-based cross-sectional survey was conducted among pregnant women admitted for delivery in Maternity and Children Hospital (MCH), Buraidah. Data were collected on socio-demographics, obstetric history, missed appointments and reasons for missing the appointment. Analysis was carried out in Statistical Package for the Social Sciences (SPSS), version 21.0 (IBM Corp., Armonk, NY). Medians with interquartile range (IQR) were presented for continuous variables and frequency and proportions for categorical variables. Logistic regression was used to assess the factors associated with the missed appointment.

Results

A total of 400 women were included in the study. About one-third (30%) of the women had missed at least one ANC appointment in their current pregnancy. The most common reasons for missing the appointments in primary care and hospitals, respectively, were: fear of infection 52% and 47%, facility not working usual 25% and 7.5%, fear of infection to child 19% and 17%. Family size and gravidity were the significant factors associated with missed appointments in our study.

Conclusions

Nearly one-third of women missed their ANC appointments during the COVID-19 pandemic. Major reasons were related to COVID-19 fear and its effect on services. This calls for proper health communication in the general population and delivering routine care with evidence-based guidelines to maintain continuity of care.

## Introduction

Coronavirus disease 2019 (COVID-19) pandemic affected the health and lives of people across the globe. This has also affected the health care services for maternal and child health [1-3]. Due to the COVID-19 pandemic, a large proportion of health care resources have been diverted from routine care delivery to the pandemic response. This diversion of resources may lead to the disruption of other essential care services [4]. This can affect the continuum of care, i.e., follow-up visits and medication supplies. Furthermore, service utilization by the population may also decline due to unavailability, fear of infection and constrained access [5]. All these factors can adversely affect the most vulnerable segment of the population, i.e., pregnant women.

Previous experiences from the Ebola outbreak have shown increased maternal and child mortality [6,7]. A similar effect has been projected for the current COVID-19 pandemic. If the essential maternal health services are not maintained, the progress so far has been made may reverse and we may experience increased morbidity and mortality in the upcoming months and years [8,9]. Maternal and child health care services need to be sustained as these require regular follow-up and a continuous supply of medicines [10]. A recent systematic review and meta-analysis reported that during the COVID-19 pandemic there was about a 38% decline in antenatal care (ANC) appointments globally [11]. Another scoping review concluded that prenatal care visits declined during the pandemic [12].

In Saudi Arabia, the public sector official gate for routine care delivery is primary health care centers (PHCCs), from which official referral to public hospitals is made for secondary care when needed. Insured clients may utilize private practice services. Out-of-pocket utilization of private practices is also common. ANC is delivered through shared care between PHCCs and hospitals while deliveries are exclusively conducted in hospitals only.

Governments, in order to control the spread of COVID-19, implemented various measures such as social distancing and lockdowns. These lockdowns could affect individuals and families in terms of their ability to access the services. On the other hand, health care systems may lose resources to sustain the uninterrupted provision of services [13]. Like any other country, Saudi Arabia was also prone to negative consequences of the COVID-19 pandemic in terms of provision of routine care besides the COVID-19 response. The country has shown remarkable progress in the maternal and child health indicators which might be affected by the COVID-19 pandemic. In order to curb the spread of infection in the country, a number of measures were taken such as mass media campaigns about COVID-19 infection and preventive strategies, promotion of social distancing, closure of educational institutions and complete lockdown. Besides these, a number of changes were also made in the health care system which included the establishment of treatment and isolation facilities for cases, the establishment of screening centers in primary care. These facilities were established by converting existing centers into special entities dealing with patients and suspected cases. Some of the primary care centers also faced closure due to a shortage of staff. Appointments of routine essential primary and acute care in these facilities were cancelled and patients were diverted to other nearby functioning PHCCs.

To the best of our knowledge, there is no published study from Saudi Arabia to look at the ANC utilization during the COVID-19 pandemic. It is therefore important to understand the impact of COVID-19 and its response on antenatal care utilization. This will help policymakers and planners to re-frame the service delivery in crises such as the COVID-19 pandemic. This study, therefore, aimed to assess the antenatal care utilization among pregnant women during the COVID-19 pandemic in the Qassim region of Saudi Arabia.

## Materials and methods

Study design and setting

This was a cross-sectional study conducted at Maternity and Children Hospital (MCH), Buraidah among pregnant women admitted for delivery from December 2020 till February 2021. MCH is the largest maternal and children health hospital in the Qassim region with about 347 beds out of which 189 beds are for women.

Sample size

The sample size was determined using the OpenEpi online sample size calculator. There is no study available which estimated the impact of COVID-19 on antenatal care utilization. A report on service utilization at different levels of care in the Gulf Cooperation Council (GCC) countries showed a decline of 70% and 50% in primary care and secondary care facilities, respectively, during the COVID-19 pandemic [14]. We assumed these figures as missed visits and used 50% for our sample size calculation as this would yield a maximum sample size. At a 95% confidence level and a margin of error of 5%, the sample size was calculated to be 385 participants.

Sampling procedure

Pregnant women admitted to MCH Buraidah for delivery were recruited consecutively from the labor room and obstetric ward. Pregnant women presenting at full term for normal vaginal delivery or caesarian section were eligible to participate. Those from outside the Qassim region were excluded.

Data collection tool and procedure

Data were collected using a structured questionnaire in Arabic. The questionnaire was developed by the research team. Variables were included based on the review of the literature. There were two sections in the questionnaire. The first section gathered information about socio-demographic characteristics and there were eight questions in this section. The second section collected data about antenatal care during the current pregnancy, missed ANC appointments and reasons for missing the appointment (Appendices). There were 20 questions in this section and an additional question that listed 12 possible reasons for missing the appointment. Data were collected by face-to-face interviews by volunteer clinicians.

Data management and analysis

Data was entered and analyzed in Statistical Package for the Social Sciences (SPSS), version 21.0 (IBM Corp., Armonk. NY). Descriptive analysis was carried to calculate frequencies and proportions for categorical variables and mean along with standard deviations for continuous variables. Median and interquartile range (IQR) were presented where data was skewed. Univariate and multivariate logistic regression analysis was carried out to find out the factors associated with missed ANC visits. Crude and adjusted odds ratios were calculated along with associated 95% confidence intervals. p-value less than 0.05 was considered significant for all inferential analyses.

Ethical considerations

The study proposal was reviewed and approved by Qassim Regional Bioethics Committee (Approval number: 1442-101360). Permission was also taken for data collection from Maternal and Child Hospital Buraidah. Verbal consent was obtained from all the participants. The confidentiality of the participants was ensured at all stages of research. Given the ongoing risk of COVID-19, the safety of data collectors was ensured by training them about preventive measures during data collection.

## Results

Socio-demographic characteristics of respondents

In this study, 400 pregnant women were recruited from maternal and child health (MCH) Buraidah. The mean age was 31.3 (±7.6) years and 202 (50.0%) were > 30 years of age. About 80% of the respondents had at least had completed high school education. The majority of them were Saudi 383 (95.8%), housewives 266 (66.5%) and lived in nuclear family 359 (89.8%). About 20% had a monthly income of less than 5000 Saudi Riyals per month. The median gravidity was 4 (IQR 3-5). The median number of living children was 3 (IQR 1-4). More than half of the mothers, 216 (54.0%) self-reported physical activity during their pregnancy and 344 (86.0%) were following the recommended healthy diet. Only 89 (22.3%) of them had a high-risk pregnancy. The study found that 60 (15.0%) of the pregnant women were infected with the COVID-19 virus and 161 (40.3%) had a history of relatives infected with COVID-19 (Table 1).

**Table 1:** Socio-demographic and health characteristics of study participants (n = 400). ^@^Nuclear family: A family where two generations, consisting of a father and mother and children or a single, possibly widow, parent and his/her children live together. ^$^Extended family: A family where three or more generations live together with both vertical and lateral extension having a single line of authority. IQR: interquartile range.

Variable	%(n)
Age	
≤30 years	49.5 (198)
> 30 years	50.0 (202)
Nationality	
Saudi	95.8 (383)
Non-Saudi	4.3 (17)
Education	
Up to intermediate school	20.5 (82)
High school	43.3 (173)
Bachelors and higher	36.3 (145)
Occupation	
Housewife	66.5 (266)
Student	6.5 (26)
Employed	27.0 (108)
Family Type	
Nuclear^@^	89.8 (359)
Extended^$^	10.3 (41)
Family size	
Mean (SD)	4.91 (1.86)
Range	(2-14)
Monthly income (Saudi Riyals)	
Less than 5000	19.8 (79)
5001 to 10000	17.0 (68)
More than 10000	63.2 (253)
Gravida	
Median (IQR)	4 (3-5)
Number of living children	
Median (IQR)	3 (1-4)
Physical activity	
Yes	54.0 (216)
No	46.0 (184)
Follow recommended diet	
Yes	86.0 (344)
No	14.0 (56)
High-risk pregnancy	
No	77.8 (311)
Yes	89 (22.3)
Ever infected with COVID-19	
No	85.0 (340)
Yes	15.0 (60)
Any of your relatives infected with COVID-19	
No	59.8 (239)
Yes	40.3 (161)
Anyone in your neighbor infected with COVID-19	
No	38.5 (154)
Yes	61.5 (246)

Antenatal care services utilization among pregnant women

The median number of ANC visits was 7 (IQR 6-8) visits. In this study, we found that the majority of the women did their antenatal care follow-up in MCH 384 (96.0%) and PHCC 257 (64.3%), while some of them utilized the antenatal care services of the private health facilities 138 (34.5%). The majority, 348 (87.0%) were satisfied with the ANC services during their last visit almost all of them 395 (98.8%) agreed about the importance of ANC. The median (IQR) waiting time for ANC consultation at the hospital was 90 (60-120) minutes while in PHCCs median (IQR) waiting time was 30 (15-45) minutes. The majority of mothers were given appointment cards 315 (78.8%). Most of the mothers 353 (88.3%) received a reminder for their upcoming ANC visit. Less than one-third 118 (29.5%) of the women used teleconsultation for ANC during this period (Table 2).

**Table 2:** Antenatal care utilization among study participants (n = 400). *Percentage is more than 100 because multiple responses were allowed. ANC: antenatal care; IQR: interquartile range; PHCC: primary healthcare centre; MCH: Maternity and Children Hospital.

Variable	% (n)
Number of ANC visits	
Median (IQR)	7 (6-8)
Place of ANC visit*	
MCH	96.0 (384)
PHCC	64.3 (257)
Private hospital/clinic	34.5 (138)
Satisfaction with ANC service during last visit	
Not satisfied	13.0 (52)
Satisfied	87.0 (348)
ANC is important	
Disagree	0.6 (2)
Neutral	0.8 (3)
Agree	98.8 (395)
Waiting time for ANC consultation at PHC (minutes)	
Median (IQR)	30 (15-45)
Waiting time for ANC consultation at hospital (minutes)	
Median (IQR)	90 (60-120)
How do you know about ANC appointment?*	
Appointment card	78.8 (315)
SMS	44.3 (177)
Phone call	13.0 (52)
Verbal	11.3 (45)
Reminder for ANC visit	
Yes	88.3 (353)
No	11.8 (47)
Used teleconsultation in the last 6 months?	
Yes	29.5 (118)
No	70.5 (282)
Did you miss ANC appointment?	
No	70.0 (280)
Yes	30.0 (120)
How many appointments did you miss? (n = 120)	
Median (IQR)	2 (1-3)

Impact of COVID-19 pandemic on ANC service utilization

The study revealed that 120 (30.0%) mothers missed or delayed their ANC appointment during the COVID-19 pandemic period, the median number of missed visits was 2 (IQR 1-3) (Table 2).

**Figure 1:**
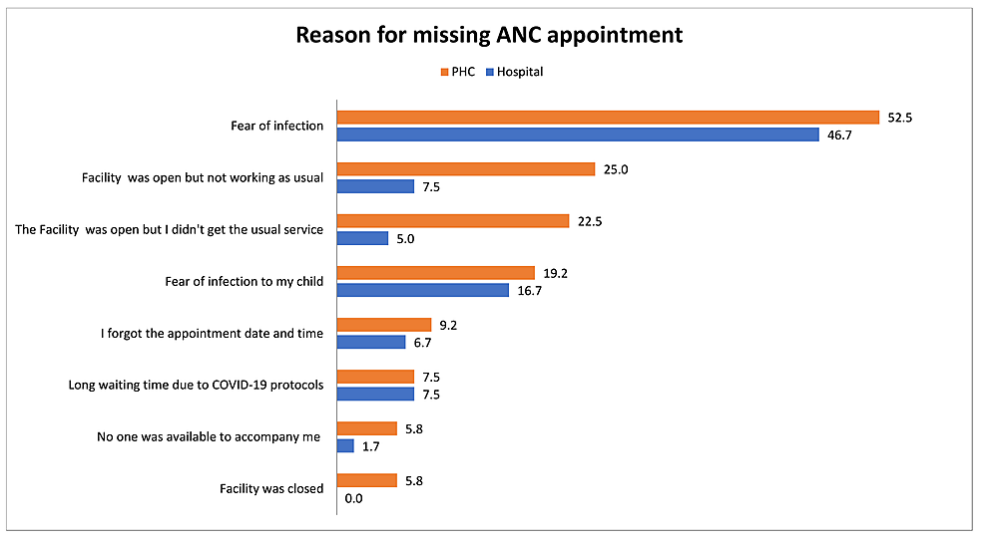
Reasons for missing/delaying ANC appointments among pregnant women. ANC: antenatal care; PHCC: primary healthcare center.

Reasons for missing ANC appointments are summarized in Figure 1. The most common reason at the hospital and PHC was fear of COVID-19 infection, 47% and 52.5%, respectively. Other common reasons at PHCCs were: the facility was open but not functioning properly and lack of usual care at PHCC 25% and 22%, respectively. In the hospital, fear of infection to the child was the second most commonly reported reason 17%.

Predictors of missed ANC appointments during COVID-19 pandemic

We explored the predictors of missed appointments using logistic regression analysis. Univariate analysis showed that education, income and following a healthy diet were significant factors. Multivariate analysis revealed that odds of missing appointment was lower among women with high school education compared to lower education level adjusted odds ratio (aOR) 0.47 (95% CI: 0.26-0.85). Family size was positively associated with a higher risk of missed appointments, aOR 1.56 (95% CI: 1.12-2.16). On the other hand, gravidity was associated with lower missed appointments, aOR 0.60 (95% CI: 0.44-0.83). Other factors were not found to be significantly associated with missed appointments (Table 3).

**Table 3:** Predictors of missed ANC appointments during COVID-19 among pregnant women in Qassim, KSA. ^©^Continuous variable. ANC: antenatal care; PHCC: primary healthcare center.

	Univariate analysis		Multivariate analysis	
Variable	Odds ratio (95%CI)	p-value	Odds ratio (95%CI)	p-value
Age				
≤30 years	1			
>30 years	0.93 (0.60-1.2)	0.727	--	--
Nationality				
Saudi	1		1	
Non-Saudi	0.30 (0.07-1.33)	0.113	0.31 (0.07-1.44)	0.137
Education				
Up to intermediate school	1		1	
High school	0.45 (0.26-0.79)	0.006	0.47 (0.26-0.85)	0.014
Bachelors and higher	0.62 (0.35-1.08)	0.091	0.58 (0.32-1.06)	0.072
Occupation				
Housewife	1			
Student	0.78 (0.32-1.94)	0.599	--	--
Employed	0.74 (0.45-1.23)	0.251		
Family Type				
Nuclear	1			
Extended	1.09 (0.54-2.19)	0.801	--	--
Family size^©^	1.00 (0.89-1.12)	0.981	1.56 (1.12-2.16)	0.008
Monthly Income (Saudi Riyals)				
Less than 5000	1		1	
5001 to 10000	0.39 (0.18-0.85)	0.017	0.50 (0.22-1.14)	0.099
More than 10000	0.83 (0.49-1.42)	0.498	1.03 (0.57-1.85)	0.921
Gravidity^©^	0.94 (0.84-1.05)	0.263	0.60 (0.44-0.83)	0.002
Number of living children^©^	0.97 (0.86-1.10)	0.698	--	--
High-risk pregnancy				
No	1			
Yes	0.95 (0.57-1.60)	0.854	--	--
Daily physical activity				
Yes	1			
No	1.20 (0.78-1.84)	0.406	--	--
Follow recommended diet				
Yes	1		1	
No	1.94 (1.09-3.46)	0.025	1.77 (0.94-3.43)	0.078
Received reminder for ANC				
Yes	1			
No	1.36 (0.73-2.60)	0.327	--	--
Teleconsultation				
Yes	1		1	
No	0.82 (0.51-1.30)	0.389	0.71 (0.43-1.16)	0.171
Waiting time at PHCC				
Up to 30 minutes	1			
More than 30 minutes	1.20 (0.76-1.88)	0.441	--	--
Waiting time at Hospital				
Up to 30 minutes	1			
More than 30 minutes	0.78 (0.42-1.45)	0.437	--	--
Infected with COVID-19				
No	1			
Yes	0.91 (0.50-1.67)	0.76	--	--
Any of your relatives infected with COVID-19				
No	1			
Yes	0.66 (0.42-1.03)	0.066	--	--
Anyone in your neighbor infected with COVID-19				
No	1			
Yes	0.75 (0.48-1.16)	0.194	--	--
Satisfaction with ANC service				
Satisfied	1		1	
Not satisfied	1.55 (0.85-2.84)	0.156	1.63 (0.82-3.26)	0.163

## Discussion

In this study, we assessed the proportion of pregnant women who missed their ANC appointments during the COVID-19 pandemic. We found that one-third of our sample missed at least one appointment. Of these, at least one quarter missed more than three appointments. Reasons for missing were mostly attributed to COVID-19. Fear of infection to oneself or to the fetus was the most reported reason, both at hospitals and PHCCs. Other reasons were mainly related to accessibility issues. Another local study conducted before COVID-19 [15], however, found that over half of surveyed women had missed at least one appointment which is higher than reported in our study. This could be due to fact that the former study had included participants from rural areas as well where ANC coverage may not be high.

ANC coverage is generally high in Saudi Arabia. A recent World Health survey for Saudi Arabia found that 80% of surveyed women reported that they had at least four ANC visits during their last pregnancy and 99% of deliveries occurred at hospitals in 2019 [16]. Early in the epidemic, pregnancy was thought to be an important risk factor for severe disease [17]. This impression had resulted in over-cautious recommendations for ANC. Saudi MOH Clinical Guidelines for Nursing & Midwifery Practice during the Coronavirus (COVID-19) Pandemic issued early in the pandemic directed midwives to "advise women to minimize the in-person antenatal visits to decrease the exposure" [18]. Furthermore, pregnant women diagnosed with COVID-19 were requested to defer ANC visits until they are cured [18]. The effect of these tough recommendations on fetal outcome was not measured by us, but a recent review had documented negative outcomes due to COVID-19 response policies [12]. Revision of epidemic response directives should be frequently conducted to minimize possible negative consequences. Similarly, public education to correct any poor evidence advice should immediately follow new solid recommendations. Quick public attitude modification is difficult, hence initial poor or low evidence advice may actually do more harm than good.

In our study, missed appointments were not significantly affected by various examined participant socioeconomic factors, lifestyle type, methods of communication with health care services, nor was consultation with the health care staff. A recent study from Saudi reported that missing ANC appointments was significantly associated with the perceived benefits of ANC, staff information and staff care [15]. Unlike the previous study from Saudi Arabia, individual and care-related factors were not found to be associated with missed appointments in our study. This might indicate the possible impact of COVID-19 on missed appointments along with other factors.

Although three-quarters of respondents reported receiving six or more ANC visits during their last pregnancy. Among those who missed their ANC appointments, one-quarter (30 women) reported missing more than two appointments, mainly due to COVID-19 health care institute policies. In Buraidah city, of the 45 active health centers, four major PHCCs were completely devoted to COVID-19 screening and two more centers were selected later for vaccine provision as well. Clients were redirected to the nearest PHCCs resulting in fragmentation of the continuity of care and overwhelming the hosting PHCCs. Husbands frequently accompany their wives during hospital visits. For ANC, this is a positive behavior for both parties, the family and health care staff. During the COVID-19 pandemic, patient companions were rejected and patients faced comfort limitations as waiting areas were either cancelled or the available seats were minimized to abide by social distancing requirements.

Information technology advancement in the Kingdom is reflected by the reported frequent participants’ use of teleconsultation and electronic appointment booking and reminders. Good utilization of these advancements was facilitated by the high educational levels of participants. It is unclear whether such measures had mitigated the negative impact of COVID-19 on ANC. Live, real office, face-to-face interaction, physical examination, office-based tests, and ultrasound are examples of missed routine ANC activities with distant ANC.

The long waiting time in hospital OPDs and missing appointments had not hindered clients’ satisfaction with care, as the majority of the participants were satisfied with the ANC services received at the last visit. It is our observation that the general public's satisfaction level with MOH PHCCs was frequently found to be good [19] despite the public voicing out their frustration through different media communication tools. 

Although this study was conducted while the pandemic was ongoing and prior to vaccine availability, a sensible proportion reported having COVID-19 themselves, many reported it among families, and more than half knew patients within their neighborhood. This reflects the widespread epidemicity despite strong control measures. A fact that deters mitigation and recovery efforts at various life activities including patient care.

Our study reflected a few aspects of routine ANC in the Kingdom. Reported ANC was a shared one as two-thirds had one or more visits to PHCCs. Private care is pacing in the Kingdom. One-third of our sample reported having some ANC at private sector clinics. As ANC is conducted at regular preplanned visits, private practices may be more favored by affluent families especially when waiting times to receive such care at public hospitals is very long as was reported here.

MCH is the only public hospital for obstetrics in Buraidah. It is also the referral hospital for all of Qassim hospitals for maternity and child care. As deliveries almost always occur at hospitals, our surveyed sample well represents the pregnant female population in Buraidah city and probably in Qassim at large, in terms of general demographic and socioeconomic status.

As the epidemic progressed, new recommendations were released and public behavior response is expected to change with changing knowledge and epidemic picture. Follow-up surveys may reflect different public responses.

## Conclusions

Near one-third of the pregnant women had missed their ANC appointments during the COVID-19 pandemic. Fear of infection and altered functioning of health facilities were common reasons. Women had to overcome obstacles and unnecessary worries dictated by protective recommendations. Judicious evidence-based guidelines should mitigate the already prevailing anxiety due to the COVID-19 pandemic instead of complicating accessibility and intensifying the already profound worries. This should be addressed by continuous mass education using various media and alternative approaches such as telemedicine to ensure a continuum of care. There is also a need for further research to explore the impact of applying such interim guidelines on maternal healthcare services.

## Appendices

Impact of COVID-19 on antenatal care utilization among pregnant women in Qassim, Saudi Arabia

Consent

We would like to invite you to participate in our research which is about impact of recently emerged diseases called Corona Virus Disease 2019 (COVID-19). In this study, researchers aim to assess the impact of COVID-19 on care of pregnant women in Buraidah, Qassim. We will ask about your antenatal care between March 2020 and August 2020. This information will help policy makers develop strategies to continue care in crises situation so that health of mother and baby is ensured. Participation in this research is totally voluntary.  Filling up this questionnaire indicates your consent to participate in the study.

**Table 4:** Section I of questionnaire about socio-demographic characteristics of the participants.

S: No:	Question	Response
1	Age	_______ years
2.	Nationality	1. Saudi
		2. Non-Saudi
3.	Education level	1. Illiterate
		2. Literate, no formal education
		3. Primary School
		4. Secondary School
		5. Graduate
		6. Masters or higher
		7. Others (please specify)
		_____________
4.	Marital status	1. Married
		2. Widowed
		3. Separated / Divorced
5.	What is your employment status?	1. Employed
		2. Student
		3. Housewife
		4. Retired
		5. Un-employed
6	Family type	1. Joint
		2. Nuclear
7	Family size (in number)	_________
			8	Monthly income	-------------- SAR

**Table 5:** Section II of questionnaire about maternal health and antenatal care utilization.

S: NO	Question	Response			Comments
1.	Gravida	_______			
2.	Number of children	_______			
3.	Do you regularly do physical activity?	1. Yes			
		2. No			
4.	Do you follow the recommended diet?	1. Yes			
		2. No			
5.	Do you think ANC visit is important for your and baby’s health?	1. Yes			
		2. No			
6.	How do you receive information about your appointment?	1. Verbally			
		2. Written on card			
		3. Mobile message			
		4. Phone call			
7.	Do you receive reminder for your ANC appointment?	1. Yes			
		2. No			
8.	Have you had a consultation on 937 for pregnancy-related care during the last 6 months?	1. Yes			
		2. No			
9.	How long do you have to wait when you visit for the ANC at PHCC?	___________minutes			
10.	How long do you have to wait when you visit for the ANC at Hospital?	___________minutes			
11.	Where did you seek ANC?	1. Primary Health Care center			
	(Select all that are applicable)	2. Private hospital			
		3. Government hospital			
12.	How satisfied are you with the services of the facility that you followed during pregnancy?	1. Very satisfied			
		2. Satisfied			
		3. Neutral			
		4. Dissatisfied			
		5. Extremely dissatisfied			
13.	How many times did you receive ANC in this pregnancy?	_________			
14.	Have you ever infected with Corona?	1. Yes			
		2. No			
15.	One of my relatives was infected with Corona at home	1. Yes			
		2. No			
16.	One of my neighbors or surrounding was infected with Corona	1. Yes			
		2. No			
17.	Have you sought to follow up your pregnancy during the COVID-19 pandemic?	1. Yes			
		2. No			
18.	Was your pregnancy high risk?	1. Yes			
		2. No			
19.	Did you miss/delayed your ANC appointment during this pregnancy?	1. Yes			
		2. No			
20.	How many visits did you miss?	________			
21.	What was/were the reason/s of missing/delaying appointment?				
	Reasons	Yes	No		
	a. Lock down				
	b. Facility was close				
	c. Facility was open but not working normal				
	d. Facility was working but I was not able to get the usual care				
	d.1 If yes, please mention the reasons				
	e. Fear of getting infection				
	f. Received call from hospital for my ANC.				
	g. Consultation through tele-medicine (937)				
	h. Long waiting due to screening for COVID-19				
	i. Forgot about the appointment				
	j. No one was available to take me to the hospital				
	k. Time of appointment was not appropriate				
	l. I was not well enough to attend				

## References

[1] Kirmani S, Saleem A (2021). Impact of COVID-19 pandemic on paediatric services at a referral centre in Pakistan: lessons from a low-income and middle-income country setting. Arch Dis Child.

[2] Wright A, Salazar A, Mirica M, Volk LA, Schiff GD (2020). The invisible epidemic: neglected chronic disease management during COVID-19. J Gen Intern Med.

[3] Chudasama YV, Gillies CL, Zaccardi F, Coles B, Davies MJ, Seidu S, Khunti K (2020). Impact of COVID-19 on routine care for chronic diseases: a global survey of views from healthcare professionals. Diabetes Metab Syndr.

[4] Sinha I, Bennett D, Taylor-Robinson DC (2020). Children are being sidelined by covid-19. BMJ.

[5] Hailemariam S, Agegnehu W, Derese M (2021). Exploring COVID-19 related factors influencing antenatal care services uptake: a qualitative study among women in a rural community in Southwest Ethiopia. J Prim Care Community Health.

[6] Suk JE, Paez Jimenez A, Kourouma M (2016). Post-ebola measles outbreak in Lola, Guinea, January-June 2015. Emerg Infect Dis.

[7] Elston J, Cartwright C, Ndumbi P, Wright J (2017). The health impact of the 2014-15 Ebola outbreak. Public Health.

[8] Roberton T, Carter ED, Chou VB (2020). Early estimates of the indirect effects of the COVID-19 pandemic on maternal and child mortality in low-income and middle-income countries: a modelling study. Lancet Glob Health.

[9] Saxena S, Skirrow H, Bedford H (2020). Routine vaccination during covid-19 pandemic response. BMJ.

[10] (2020). World Health Organization: COVID- 19: Operational guidance for maintaining essential health services during an outbreak: Interim guidance. Geneva, Switzerland. https://apps.who.int/iris/handle/10665/331561.

[11] Townsend R, Chmielewska B, Barratt I (2021). Global changes in maternity care provision during the COVID-19 pandemic: a systematic review and meta-analysis. EClinicalMedicine.

[12] Kotlar B, Gerson E, Petrillo S, Langer A, Tiemeier H (2021). The impact of the COVID-19 pandemic on maternal and perinatal health: a scoping review. Reprod Health.

[13] McKee M, Stuckler D (2020). If the world fails to protect the economy, COVID-19 will damage health not just now but also in the future. Nat Med.

[14] (2020). Half of GCC's regular healthcare on hold due to Covid-19. https://www.consultancy-me.com/news/2716/half-of-gccs-regular-healthcare-on-hold-due-to-covid-19..

[15] Alanazy W, Brown A (2020). Individual and healthcare system factors influencing antenatal care attendance in Saudi Arabia. BMC Health Serv Res.

[16] (2021). World Health Survey: Saud Arabia. (2019). Accessed: August 15. https://www.moh.gov.sa/en/Ministry/Statistics/Population-Health-Indicators/Documents/World-Health-Survey-Saudi-Arabia.pdf..

[17] Phoswa WN, Khaliq OP (2020). Is pregnancy a risk factor of COVID-19?. Eur J Obstet Gynecol Reprod Biol.

[18] (2021). Clinical Guidelines for Nursing & Midwifery Practice during the Coronavirus (COVID-19) Pandemic. https://www.moh.gov.sa/en/Ministry/MediaCenter/Publications/Documents/Clinical-Guidelines-for-Nursing-Midwifery-Practice-during-COVID-19-Pandemic.pdf..

[19] Senitan M, Alhaiti AH, Gillespie J (2018). Patient satisfaction and experience of primary care in Saudi Arabia: a systematic review. Int J Qual Health Care.

